# The Proteolytic Regulation of Virus Cell Entry by Furin and Other Proprotein Convertases

**DOI:** 10.3390/v11090837

**Published:** 2019-09-09

**Authors:** Gonzalo Izaguirre

**Affiliations:** College of Dentistry, University of Illinois at Chicago, Chicago, IL 60612, USA; goniza@uic.edu

**Keywords:** furin, proprotein convertases, proteases, protease inhibitors

## Abstract

A wide variety of viruses exploit furin and other proprotein convertases (PCs) of the constitutive protein secretion pathway in order to regulate their cell entry mechanism and infectivity. Surface proteins of enveloped, as well as non-enveloped, viruses become processed by these proteases intracellularly during morphogenesis or extracellularly after egress and during entry in order to produce mature virions activated for infection. Although viruses also take advantage of other proteases, it is when some viruses become reactive with PCs that they may develop high pathogenicity. Besides reacting with furin, some viruses may also react with the PCs of the other specificity group constituted by PC4/PC5/PACE4/PC7. The targeting of PCs for inhibition may result in a useful strategy to treat infections with some highly pathogenic viruses. A wide variety of PC inhibitors have been developed and tested for their antiviral activity in cell-based assays.

## 1. Introduction

The regulation of viral cell entry by proteases is a control mechanism common among viruses (Table 1). The proteolytic processing of viral proteins is often required for virus maturation and infectivity. A critical group of host-cell proteases exploited by a variety of viruses is the family of proprotein convertases (PCs), which includes furin, PC4, PC5, PACE4, and PC7 [1,2]. Although other types of proteases besides PCs can also perform the proteolytic maturation of viruses, it has been observed that when PCs process viral proteins, some viruses become comparatively more infective and pathogenic. Most of the research done on the maturation of viruses by PCs has focused on furin. However, there is evidence of the involvement of other PCs in the regulation of virus maturation [3]. The scattered information about the role of PCs in the life cycle of a wide variety of viruses [4,5], in addition to the new developments on PC activity regulation and reaction specificity [6,7,8,9], calls for an effort to integrate this knowledge, analyze the relevance of PCs in the pathogenicity of viruses, and evaluate the feasibility of inhibiting PCs as a sound strategy for antiviral therapy. This review will discuss the importance of differences of PC reactivity and selectivity, and the PC gene expression profile of infected cells, in determining virus infectivity and tropism.

The proteolytic maturation of viruses by PCs generally involves the processing of proteins localized on the surface of viral particles, either of non-enveloped or enveloped viruses [10]. The cleavage of the surface viral proteins mostly occurs inside the host cells during virus morphogenesis and before egress, although cleavage by the target-cell PCs can occur extracellularly or during cell entry with some viruses. The proteolytic processing by PCs promotes binding and fusion of viral particles to target cells.

**Figure 1:**
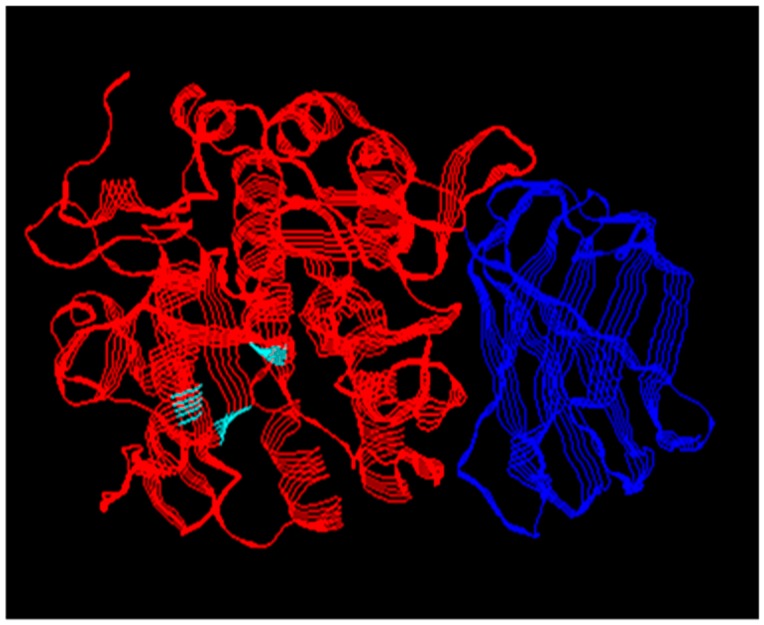
X-ray crystal structure of human furin. The PC catalytic domain (red) shared by all PCs has the structural fold typical of the subtilin family of serine proteases. The position of the catalytic triad residues is marked in cyan. The P domain (blue) regulates catalytic activity. The PCs differ in their additional domains. PDB ID code 5JXH.

PCs are eukaryotic serine proteases classified in the MEROPS Peptidase Database within the S8B family. Furin, PC4, PC5, PACE4, and PC7 are part of the Kexin-like subfamily of PCs and localize to the organelles of the constitutive protein secretion pathway [1]. These PCs perform the proteolytic post-translational modification of a large variety of peptides and proteins in the trans-Golgi network, endosomes, and pericellular environment, and are critical regulators of central cellular processes, such as growth, proliferation, and differentiation [11,12,13]. The gene expression profile of the Kexin-like PCs is cell-type dependent, but most cells express some or all of them, except for PC4 whose expression is restricted to cell types in the testes, ovaries and the placenta. PCs are large-size multidomain proteins composed of conserved catalytic and regulatory P domains that share 50–65% amino acid sequence homology (Figure 1). Furin, PC5, and PC7 are type I membrane-bound proteins, and furin and PC5 can be shed extracellularly; in contrast, PACE4 is a secreted protein. Kexin-like PCs cleave their substrates at sites specified by a motif composed of P4Arg—P3X—P2X—P1Arg—P1′X, where X is any amino acid residue, and cleavage occurs between the P1Arg and P1′X residues. This sequence motif is found in many viral surface proteins and determines cleavage by PCs [3]. The Kexin-like PCs are divided into two specificity groups, one represented by furin, and the other by PC4/PC5/PACE4/PC7 [9]. Furin is more reactive than the other PCs, and the differences in reaction specificity between the two groups are based on active-site and exosite determinants of reactivity.

Kexin-like PCs are considered potential pharmacological targets for the treatment of viral infections by blocking virus maturation and infectivity. Other uses for the targeting of these PCs include the inhibition of the activation of bacterial toxins such as *Shiga*, anthrax, *Clostridium*, *Pseudomonas*, and diphtheria; and also, for the treatment of degenerative diseases such as metastatic cancer, Alzheimer’s, and osteoarthritis [2,4,5]. The only known natural inhibitors of PCs are serpins, which are slow-binding type inhibitors that form covalently-linked inhibitory complexes with their target proteases [14]. Serpin B8, currently the only PC inhibitory serpin identified in vertebrates, has higher specificity for furin than for PC4/PC5/PACE4/PC7 [9,15,16]. More PC inhibitory serpins have been characterized in other organisms as well [17,18,19]. A variety of synthetic PC inhibitors have been developed based on small molecules, peptides and their mimetic derivatives, and larger proteins [5,20]. However, the main obstacle for their therapeutic use has been their toxicity, and their lack of PC selectivity. An important research tool is the PC inhibitor α1PDX, which is a derivative of the serpin α1-antitrypsin with an engineered PC cleavage site motif at its reactive loop [21]. This engineered serpin inhibits all the Kexin-like PCs with the same specificity. More recently, we developed two α1PDX-serpin B8 chimeras that selectively target each of the two PC specificity groups. One is α1ORD that specifically inhibits furin, and the other is α1MDW that specifically inhibits PC4/PC5/PACE4/PC7 [8,9].

The literature on the proteolytic processing of viral surface proteins by PCs and the role that PCs play on the maturation of viruses will be reviewed, and finally, the development of PC inhibitors and their antiviral properties will be discussed.

## 2. Papillomaviruses

The human papillomavirus (HPV) infects the basal cells of stratified epithelium, and virion replication depends on the infected basal cells progressing into differentiated squamous cells. HPV infects by reaching the lower layers of the stratified epithelium through micro-wounds in the tissue. There, the viral particles bind to heparin sulfate proteoglycan receptors localized either on the extracellular matrix of the basement membrane or the cell surface [22,23]. HPV particles are constituted by a naked nucleocapsid, and work done with pseudovirion particles has suggested that conformational changes in the nucleocapsid proteins L1 and L2, that are induced upon binding of the virus to cell-surface proteoglycans, prime L2 for cleavage by extracellular or pericellular PCs at the Arg12 residue [24]. The cleavage of L2 modifies the conformation of the coat proteins and allows the virion to engage another receptor, and that leads to cell internalization and infection [25,26]. The inhibition of the target-cell PCs blocks HPV infection, but the treatment of the pseudovirion particles with furin beforehand bypasses the inhibition. In contrast, the cleavage of live native HPV16 virions by PCs occurs during virion morphogenesis, so infectivity becomes independent from the target-cell PCs [27,28,29]. Also, the proteolytic processing seems to be HPV-type dependent, as evidenced by the native HPV18 virions being poorly processed during morphogenesis, and their infectivity being mostly dependent on the PCs of the target cells [27].

L1 and L2 in HPV16 and HPV18 contain more PC cleavage site motifs besides the commonly studied L2-Arg12. Two PC cleavage site motifs, one at L1-Arg74 and the other at L2-Arg305, are conserved in many HPV types (Table 2). Mutagenesis of the L1-Arg74 site has been reported to affect pseudovirion morphogenesis [30]. The L2-Arg305 site is located in a region of L2 involved in the regulation of retrograde trafficking of the L2-viral genome complex from the trans-Golgi network and into the nucleus [31,32]. Surprisingly, low-risk HPV types have Lys at the L1-74 position instead of the Arg of high-risk types (Table 2). If cleavage at the L1-74 site is required for virus morphogenesis, low- and high-risk types may use different proteases, unless their morphogenesis is different. If the cleavage at the L1-Arg74 and L2-Arg305 sites takes place during cell entry, the cleavage sites may be hidden inside the folded protein and protected from being accessed by the PCs in the intact virion. However, the virions undergo controlled unfolding during entry and trafficking, so that the PC cleavage sites may become exposed and cleaved along their cell internalization route.

The potential diverse expression of the PC genes in keratinocytes at different anatomical sites of HPV infection may contribute to the restricted cell tropism by HPV types, which is especially different between the skin and mucosal types [33]. HPV16 is commonly found in the stratified epithelium of the ectocervix and tonsilar crypts, and HPV18 is mainly found in the glandular epithelium of the endocervix. Their differences in PC reactivity may play a role in determining their particular tropism.

## 3. Herpesviruses

The envelope glycoprotein B (gB) is the most conserved protein among all herpes viruses, and its function is to regulate virus to cell membrane fusion. gB is synthesized as a precursor protein, and PCs cleave it at a loop, which is located in domain II of the ectodomain and at a distance from the fusion loop at domain I. The cleavage site loop is highly variable in length and amino acid sequence among herpes viruses (Table 3). The cleavage of gB by PCs has been demonstrated [34]. Most herpes viruses have at least one PC cleavage site motif in the cleavage loop, although, HHSV1 is an exception by having no PC motifs at all. In contrast, other viruses have more than one PC cleavage site, which may be cleaved sequentially [35]. The experimental inactivation of the PC cleavage site of several herpes viruses did not severely affect viral cell entry into cells growing in vitro; however, the lack of PC cleavage reduced virus spread and replication in vivo [36,37]. The cleavage of gB promotes virus-to-cell and cell-to-cell fusion [36,38]. Although much is still needed to consolidate our knowledge of the cleavage of gB by PCs, there is no doubt that the presence of PC cleavage site motifs in gB is the result of selective evolutionary pressure [39].

## 4. Flaviviruses

Two proteins predominate in the envelope of flaviviruses, prM, and glycoprotein E [40]. The association between the two proteins (prM-E) in the immature virus changes upon cleavage of prM by PCs during egress. The pr segment is removed to render the mature virions (M-E) (M-E) [41]. All flaviviruses contain a PC cleavage site motif at the pr-M junction (Table 4).

A peculiar case is the maturation of the Dengue virus (DENV). Its proteolytic processing is known to be very inefficient, and virions are produced in the prM-E form in high proportion. It was initially suspected that maturation might not be necessary for infectivity but later demonstrated that it is indeed needed [42]. The inefficient maturation of the DENV agrees with studies that show that anti-prM antibodies represent a significant proportion of the immune response to DENV and that these antibodies are responsible for the development of antibody-dependent enhancement (ADE) of infection in individuals suffering from recurrent DENV infections [43]. These observations suggest that the DENV PC reactivity is weaker compared to that of other flaviviruses, which seem to mature more efficiently.

The PC site sequence alignment presented in Table 4 shows that the four DENV types have Asp or Glu residues at the P3 position of the cleavage site, compared to Ser or Thr in most other flavivirus sequences, including that of the zika virus (ZIKV). Acidic residues at this position in the substrate sequence are detrimental to reactivity with PCs [9]. Based on these differences, it is expected that DENV reacts with a dramatically lower reactivity toward the PCs compared to other flaviviruses and that higher rates of PC reactivity align with the strong virulence and broad cell tropism observed with other flaviviruses such as [44]. Therefore, it is not surprising that ZIKV can even reach the fetus and remain in bodily fluids of asymptomatic patients for more extended periods when compared to DENV [45]. Variations of the PC gene expression profile may be a key factor determining the difference of tropism to testes between ZIKV and DENV. PC4 is the primary PC expressed in testes [46]. A vigorous reactivity of ZIKV with PC4 would explain why the testes suffer the highest loads of ZIKV compared to other organs and the sexual transmission of the virus. The viral PC reactivity and the cell PC gene expression profile both probably play a role in determining the cell tropism differences observed with flaviviruses [47,48,49].

## 5. Togaviruses

Viruses of the genus *Alphavirus* like Chikungunya (CHIKV), Semliki forest (SFV), Sindbis, and Ross River, all are arboviruses structurally related to flaviviruses [50]. Their glycoprotein precursor E3E2 is cleaved by PCs in order to regulate its interaction with the glycoprotein E1, which promotes virus to cell fusion and infection [51,52]. The information available about the processing of togavirus proteins by PCs is scant, but it reveals the existence of amino acid sequence variability in the PC cleavage sites between the CHIKV Asian and African strains, and that this variability probably determines the observed differences of PC selectivity [52].

## 6. Coronaviruses

The family of coronaviruses includes viruses of relevance to human and veterinary health. Like other enveloped viruses that rely on surface glycoproteins for binding and fusion, coronaviruses have the Spike (S) protein, which is cleaved by proteases during virion biosynthesis, as well as during entry into target cells [53]. The proteolytic regulation of coronaviruses is probably one of the best-studied systems, and a complete picture of the regulatory system mechanism has been developed compared to other families of viruses that are less well-studied. The general principles of the proteolytic regulatory mechanism of coronaviruses based on the accumulated evidence include: (1) these viruses are regulated by a variety of proteases, (2) the protein S is cleaved sequentially at two cleavage sites, (3) viruses can quickly adapt to the proteolytic environment of the infected cells, and (4) the compatibility between the cleavage site-specificity and cell protease expression profile determines the cell and tissue tropism and pathogenicity of the virus.

Furin is not the only protease that regulates the function of the coronavirus fusion protein. Other proteases, such as the membrane-bound TMPRSS, the lysosomal cathepsins, elastase, and coagulation factor Xa have also been implicated [54,55]. Protein S is cleaved at the S1–S2 junction during biosynthesis to separate the two major domains of the protein. The S1 domain is involved in receptor binding, and the S2 domain mediates the fusion step of the cell entry mechanism. During cell entry, the cleavage at S1–S2 primes S for the second cleavage at the S2′ site [56,57,58,59]. In many coronaviruses, the S1–S2 cleavage seems to be dispensable; however, the cleavage at S2′ is not. The cleavage at S2′ has been suggested to serve as a virulence marker [53]. Predictions of the furin/PC reactivity, based on the amino acid sequence surrounding the cleavage site, have been made based on computer algorithms [13]; however, the dependency of furin/PC reactivity on the conformation of the substrate and exosites lends uncertainty to those predictions.

The highly virulent MERS-CoV (Middle East respiratory syndrome coronavirus) is the only natural virus known to have PC cleavage site motifs at both the S1–S2 and S2′ sites. Other viruses with two PC sites are the result of laboratory selection by their serial passage in cell lines in vitro, one such virus being the infectious bronchitis virus IBV-Beaudette strain [60]. MERS-CoV has an expanded tropism compared to other coronaviruses, so it is considered polytropic [58]. Only the S2′ site in SARS-CoV (severe acute respiratory syndrome coronavirus) has a PC cleavage site motif [54,55]. The fact that MERS-CoV and SARS-CoV are highly pathogenic, and that IBV-Beaudette is apathogenic is in line with these viruses reacting with proteases other than the PCs [61,62]. TMPRSS2 promotes SARS-CoV and MERS-CoV infection in vivo [63].

The engineering of PC specificity at the cleavage sites of coronavirus S proteins can modify the virus tropism and virulence [64,65]. The conversion of a monobasic cleavage site into a polybasic site not only makes the virus susceptible to PC cleavage but also increases the chance of cleavage by other proteases that target single arginine residues, so it is not surprising that MERS-CoV is so pathogenic. Because coronaviruses are adapted to the different proteolytic environments of the many cell types they infect, each virus may be activated by a specific set of proteases. It is crucial to define the protease cleavage specificity of viruses that impact human or animal health. The use of the PC inhibitor, dec-RVKR-cmk, has created some controversy as sometimes the inhibitor is used in excessive concentrations. The inhibitor binds PCs with a very high affinity, at low nM concentrations; it slowly forms covalent complexes with the enzymes, so it inhibits PCs in a stoichiometric manner. In our hands, treating cells with this inhibitor at a concentration of 1 µM is enough to block HPV16 cell entry completely. Concentrations up to 100 µM reported in some studies should not be considered PC-specific; such high inhibitor concentrations most probably inhibit other proteases besides PCs [58,64].

## 7. Retroviruses

**Figure 2:**
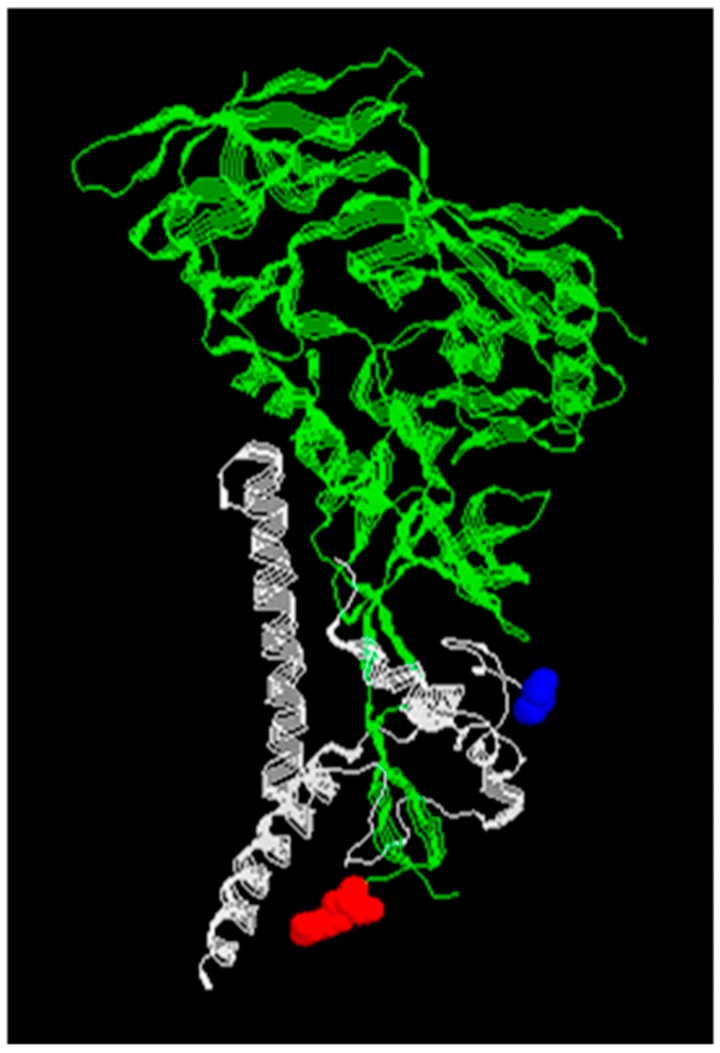
X-ray crystal structure of the HIV-1 envelope pg160 glycoprotein monomer. The fusion machine is composed of three gp160 monomers which are divided into N-terminal gp120 (green) and C-terminal pg41 (gray). Residue in red denotes the end of gp120 and residue in blue the beginning of gp41 after PC cleavage. PDB ID code 6MTJ.

Medically relevant retroviruses of the *Retroviridae* family have also been studied concerning their proteolytic regulation. The most studied viruses are the bovine and murine leukemia viruses, which are related to the human T-lymphotropic viruses. Their envelope glycoproteins are cleaved by furin and other PCs [66]. Like the coronaviruses, leukemia virus glycoproteins are cleaved twice. PCs perform the first cleavage, which induces conformational changes and disulfide isomerizations that prime the protein for further proteolysis [67,68]. The second proteolytic event is performed by a viral protease that fully activates the glycoprotein [69,70]. The human immunodeficiency virus (HIV) Env glycoprotein gp160 precursor is cleaved by furin during biosynthesis into gp120 and gp41 in the trans-Golgi network (Figure 2). gp120 is further processed by furin into gp77 and gp53 after leaving the TGN [71,72]. The Env glycoprotein is the only antigenic HIV protein, and furin cleavage-independent forms stabilized in the native form have been produced for vaccine development purposes [73]. Interestingly, a polybasic region located upstream from the PC cleavage site at the gp120/gp41 junction was shown to bind heparin and promote cleavage [74].

## 8. Hepadnaviruses

The duck hepatitis B virus (DHBV) has been used as a model to study the hepatitis B virus (HBV). The proteolytic events that regulate the cell entry mechanism of this hepadnavirus have not attracted much attention, but there is evidence of the cleavage of the envelope proteins by PCs [75].

## 9. Filoviruses, Bunyaviruses and Arenaviruses

Single-stranded negative-sense RNA viruses of the *Filoviridae* and *Arenaviridae* families and the new-order *Bunyavirales* are the causative agents of lethal hemorrhagic fever diseases. Despite the seriousness of the health threat these viruses represent, the information about the proteolytic regulation of their entry mechanism is scarce. The envelope glycoproteins of the Ebola (EBOV) and Marburg (MBGV) viruses are processed by furin into two disulfide-linked subunits [76,77,78]. Except for the Reston strain that has no PC cleavage site motifs, all other EBOV strains have one; the Reston strain is less pathogenic than the other EBOV strains [76]. The glycoprotein of MBGV has two PC cleavage site motifs that do not agree in their amino acid sequence and position compared to the single PC site in the EBOV protein [78]. The cleavage by furin seems to be dispensable because the elimination of the PC site in the EBOV protein does not affect the virus replication in cultured cells or the disease progression in experimental animals [79,80,81]. EBOV requires further proteolytic processing of the glycoprotein binding domain by endosomal cathepsins in order to gain binding activity [82,83,84]. Filoviruses are different from other viruses in that they require additional factors or modifications of the glycoprotein in order to gain infectivity [85,86]. The Crimean-Congo hemorrhagic fever bunyavirus (CCHFV) glycoprotein is processed by furin and the proprotein convertase SKI-1, a PC of the pyrolysin-like type and also known as S1P, which has a cleavage site specificity different from the polybasic specificity of the Kexin-like PCs [87,88]. The cleavage by furin is not essential, but inactivating the cleavage site slows down virus replication [89,90]. The lymphocytic choriomeningitis (LCMV) and the Lassa (LASV) arenaviruses are known to also require SKI-1 activity for the cleavage of their envelope glycoproteins [89,90].

## 10. Paramyxoviruses

The *Paramyxoviridae* is a diverse family of viruses, and a variety of proteases activate their fusion proteins. Some paramyxoviruses are highly pathogenic. Single proteolytic processing of the fusion protein occurs for most of these viruses. PCs perform the cleavage in the parainfluenza and the measles (MV) viruses [91,92,93]. There are several serotypes of the avian paramyxoviruses (APMV). The glycoprotein of the highly pathogenic APMV-1, or Newcastle disease virus (NDV), is cleaved by furin, and the proteins of other serotypes are cleaved by undetermined trypsin-like proteases [94,95]. The mutation of the trypsin-like sites into PC site motifs made the viruses replicate faster in cell culture, but they remained non-virulent in vivo [96,97,98]. Conversely, the transformation of the PC cleavage site of the virulent APMV-1 strain into a trypsin-like site induced the virus to become highly attenuated [99]. The pathogenic respiratory syncytial virus (RSV) is unique among paramyxoviruses in that its glycoprotein is cleaved at two sites by PCs [100]. The first cleavage takes place before the virus enters the target cells, and the second occurs after entry into the endosomes [101]. Furin does not activate the lethal Nipah (NiV) and Hendra (HeV) viruses for entry; instead, the viruses depend on endosomal cathepsins [102,103]. These viruses produce systemic infections in several different hosts. The glycoprotein of the Sendai virus (SeV) requires the participation of the homologous attachment protein hemagglutinin-neuraminidase (HN), which binds the cell surface sialic acid receptors. SeV glycoprotein has only one trypsin-like site, but by replacing it with the two RSV PC sites, the dependency on HN for infection is reduced [104].

## 11. Orthomyxoviruses

**Figure 3:**
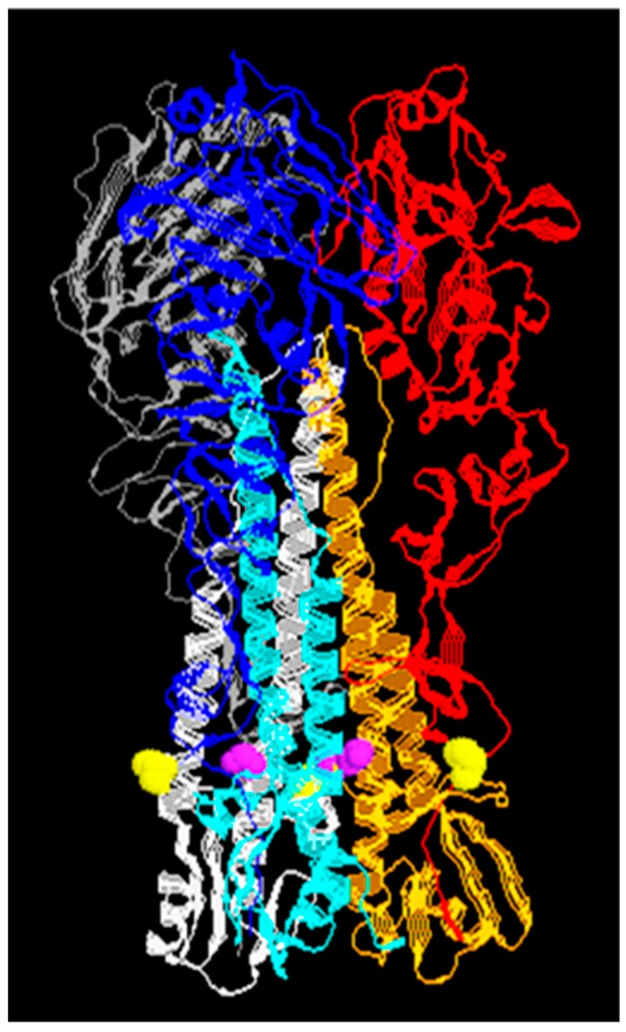
X-ray crystal structure of the HA trimer from the influenza virus A type H5. Each HA monomer is divided into HA_1_ (red, blue, and gray) and HA_2_ (oarange, cyan, and white, respectively) subunits after the PC cleavage denoted by residues at the end HA_1_ (yellow) and the beginning of HA_2_ (purple). PDB ID code 2IBX.

The influenza viruses cause respiratory disease and occasional pandemics. The virus envelope contains two glycoproteins, hemagglutinin (HA) and neuraminidase (NA). Both proteins contribute to the virus pathogenicity and the cleavage of HA_0_ precursor into HA_1_ and HA_2_ by the host cell PCs is a significant contributor of virulence for avian influenza (Figure 3). The extent and diversity of the cellular proteolytic activity is also an essential factor determining pathogenicity, spread, and tropism of the influenza virus [105,106]. There are 16 HA types, but H1 and H2 are the most commonly present types in seasonal human infections, other types are found in birds. The pandemics of 1957 and 1968 were caused by the H2N2 and H3N2 strains, respectively. The proteolytic cleavage of HA occurs in a loop that varies in length and amino acid sequence depending on the strain (Table 5). The loop usually contains one Arg residue that determines cleavage by trypsin-like proteases. The cleavage can occur during synthesis, after release or before entry, and may depend on different proteases. The highly pathogenic virus strain responsible for the Spanish 1918 influenza pandemic was of the H1N1 type with only one Arg residue in its cleavage loop. Two proteases highly expressed in the respiratory tract, especially in the lungs, TMPRSS2 and TMPRSS4, were shown to cleave the 1918 influenza HA [107]. HAT is a protease expressed in the airways, mostly in the larynx but not in lungs. It is also capable of activating influenza viruses [108].

Multibasic cleavage sites in HA arise by single substitution mutations like in the case of some H9N2 types, or by insertions that result in longer loops, as observed with the highly pathogenic H5 and H7 types. Viruses that acquire multibasic cleavage sites become independent of trypsin-like proteases. In low-pathogenic H9N2 strains carrying the cleavage site motif, R-S-K-R, cleavage is not performed by PCs but by matriptase, which recognizes the same cleavage site motif of PCs and determines the nephrotropism of the virus [109]. However, H9N2 can become reactive with PCs by the removal of a glycosylation site near the cleavage site [110]. The long and multibasic loops in some H5 and H7 strains are highly reactive with furin [111,112,113,114]; this reactivity leads to high pathogenicity that causes systemic infections [115,116]. An outbreak of a highly pathogenic avian H5N1 strain that infected humans occurred in 1997 in Hong Kong. In some highly pathogenic H5 and H7 types that have the K-K-K-R motif, cleavage is carried out by the ubiquitous protease MSPL and its splice variant MTPRSS13, which are also capable of cleaving at the PC cleavage site motif [117].

## 12. Antiviral PC Inhibition

The search for effective PC inhibitors centers into finding the inhibitor with the best characteristics of specificity, stability, and bioavailability [118]. Most PC inhibitors reported have been developed against furin. Although these inhibitors are of high PC specificity, many of them still lack proper characterization of their PC selectivity. Knowing the PC selectivity of an inhibitor is a critical issue as PCs differ in substrate specificity, and viruses can be PC-selective. Synthetic PC inhibitors come in several forms, from small molecules identified by high-throughput screening [119,120,121,122]; to peptide substrates [123,124], or viral cleavage sites [125]; peptide mimetic derivatives that add unnatural amino acids [126,127,128,129,130,131,132,133,134,135]; cyclic peptides [136]; polyarginine [137,138,139]; and larger engineered proteins like the leech eglin C [140], turkey ovomucoid [141], α2-macroglobulin [142]; and the engineered serpin α1-PDX [21]. Peptide derivatives seem more efficient at producing high-affinity PC inhibitors compared to small molecules [118]. Due to the high density of negative charges at the PC active site, highly basic peptides show strong specificity and bioavailability, but may also be highly toxic [133]. Larger proteins are poised to become the most effective PC inhibitors as they offer better opportunities to build specificity and selectivity compared to small molecules. They can also be made bioavailable through a variety of routes. Several of these PC inhibitors have been confirmed to have antiviral activity in cell-based assays of viral propagation (Table 6).

## 13. Conclusions

The ubiquitous presence of furin and related PCs throughout the cells of the body makes these proteases vulnerable to being exploited by viruses. The location of furin and related PCs in the vesicles of the constitutive protein secretion pathway, where viruses are assembled during morphogenesis or disassembled during cell entry, explains why a diversity of virus types have evolutionarily converged to depend on PCs. Viruses also use other types of proteases for the proteolytic regulation of the binding and fusion functions; however, proteases are restricted to specific cell types, which limits the range of the viral infection, so when some viruses mutate and acquire PC reactivity, they may expand their cell tropism and become more pathogenic.

The targeting of PCs for inhibition as an antiviral strategy is a sound possibility. Probably the major advantage of this approach is that by not targeting a viral component or function, it reduces the chance of producing resistance. The main drawback is the ubiquitous distribution of PCs and the potential toxicity and secondary effects that their inhibition may cause. In consequence, it is essential to know the virus PC selectivity and to have PC inhibitors that are selective for one of the two PC specificity groups, furin or PC4/PC5/PACE4/PC7 [9].

## Figures and Tables

**Table 1:** Families of pathogenic viruses that are PC dependent. Many families of viruses exploit the host-cell PCs to regulate their cell entry mechanism.

Familiy	Virus	Capsid	Genome
*Papillomaviridae*	HPV	Naked	Circular dsDNA
*Herpesviridae*	Herpes, Cytomegalovirus, Epstein-Barr, Varicella-zoster	Enveloped	Linear dsDNA
*Flaviviridae*	Dengue, Zika, Yellow fever, West Nile	Enveloped	Linear ssRNA^+^
*Togaviridae*	Chikungunya, Semliki forest	Enveloped	Linear ssRNA^+^
*Coronaviridae*	MERS	Enveloped	Linear ssRNA^+^
*Retroviridae*	HIV, Leukemia viruses	Enveloped	Linear ssRNA^−^-RT
*Hepadnaviridae*	Hepatitis B	Enveloped	Linear ssDNA^−^-RT
*Filoviridae*	Ebola, Marburg	Enveloped	Linear ssRNA^−^
*Paramyxoviridae*	Measles, RSV, Newcastle disease, Nipah	Enveloped	Linear ssRNA^−^
*Orthomyxoviridae*	Avian influenza H5N1	Enveloped	Linear ssRNA^−^

**Table 2:**
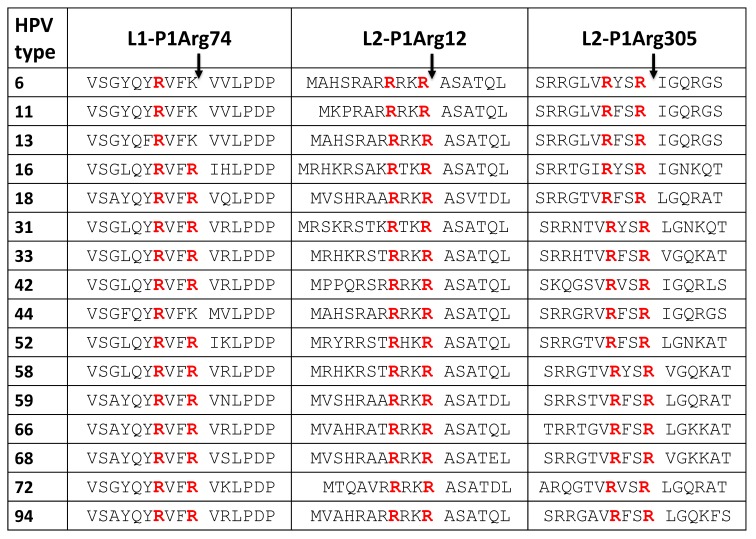
PC cleavage site motifs in the coat proteins L1 and L2 of HPV types. The cleavage site numbers correspond to those in the HPV16 sequences. The P4Arg and P1Arg residues are denoted in red.

**Table 3:**
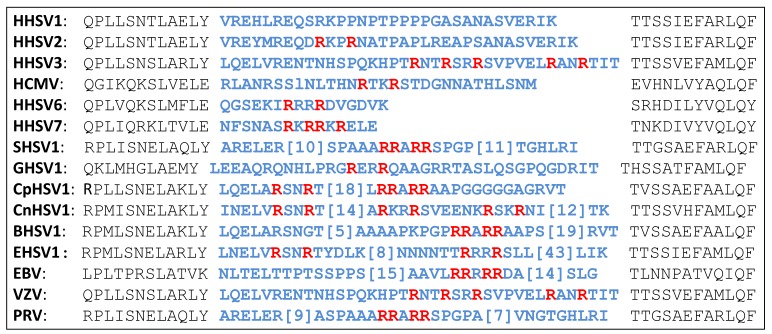
PC cleavage site motifs in the glycoprotein B of herpes viruses. A highly variable loop in the viral glycoprotein B (residues in blue) contains PC cleavage site motifs (P4Arg and P1Arg residues are red). HHSV, human herpes simplex virus; SHSV, suid herpes simplex virus; GHSV, gallid herpes simplex virus; CpHSV, caprine herpes simplex virus; CaHSV, canid herpes simples virus; BHSV, bovine herpes simplex virus; EHSV, equide herpes simplex virus; EBV, Epstein-Barr virus; VZV, varicella-zoster virus; HCMV, human cytomegalovirus; PRV, pseudorabies virus.

**Table 4:**
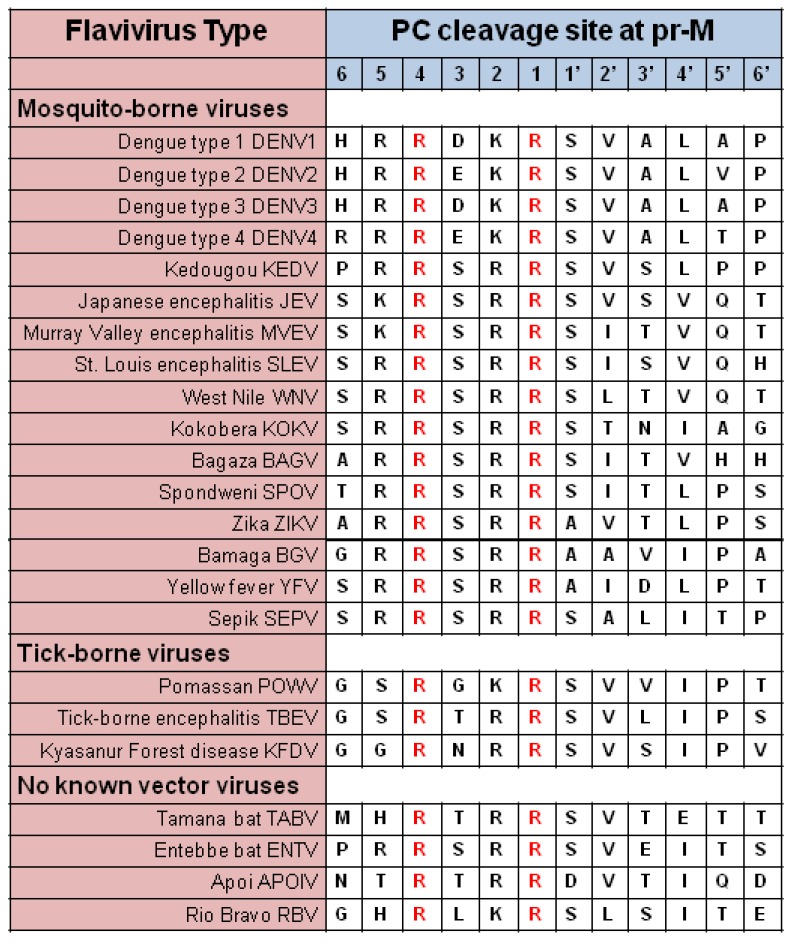
Alignment of flavivirus PC cleavage sites.

**Table 5:**
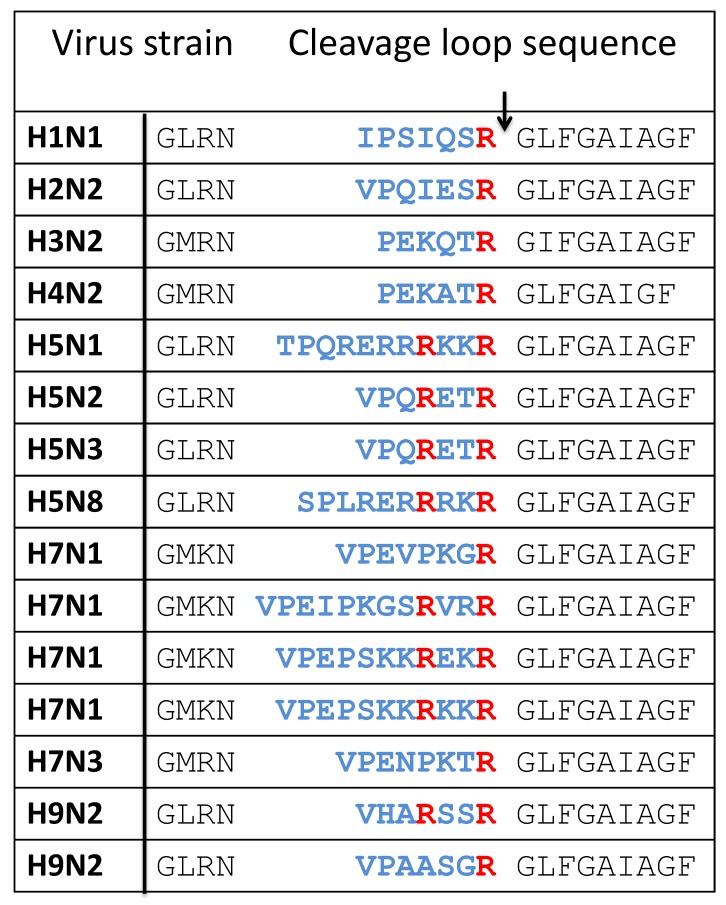
Cleavage loop in the HA protein of influenza viruses. The variable region is blue with the arrow denoting the cleavage site. The P4Arg and P1Arg residues are colored red.

**Table 6:** Inhibitors of PCs tested for their antiviral activity in cell-based assays of viral propagation.

Inhibitors	Viruses	References
SKI-1 inhibitors	LCMV, LASV	[89,90]
Peptides and peptidomimetics	CHIKV, SFV WNV, DENV H5 and H7 influenza	[52,134] [133,135] [123,128,129,131,132]
Polyarginines	HIV	[143]
Macrocyclic peptides	RSV	[136]
α1PDX	MV EBOV, MBGV HCMV	[92] [144] [145]
